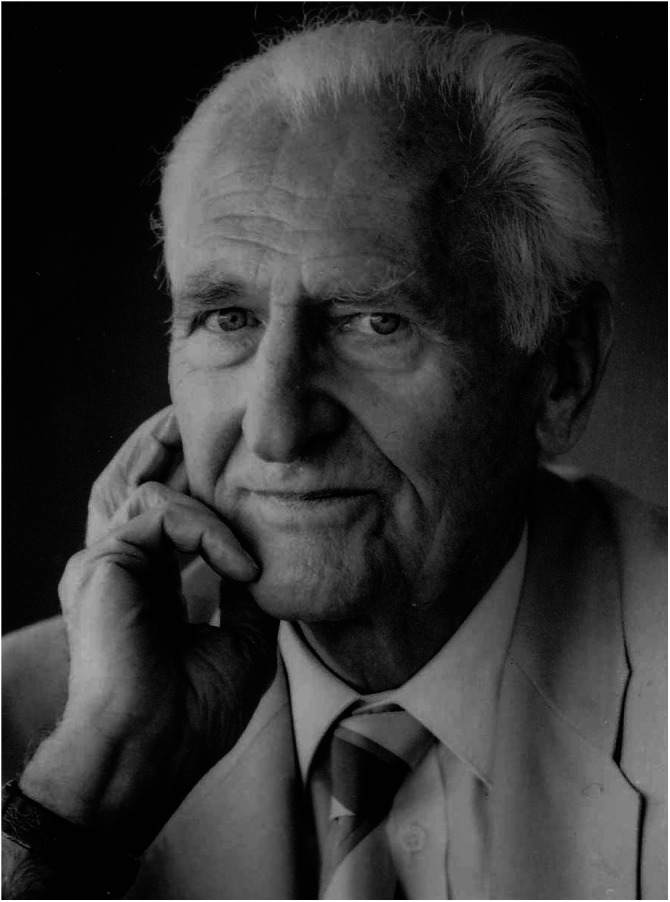# Sir Richard Doll, epidemiologist – a personal reminiscence with a selected bibliography

**DOI:** 10.1038/sj.bjc.6602812

**Published:** 2005-10-25

**Authors:** Leo Kinlen

The death of Richard Doll on 24 July 2005 at the age of 92 after a short illness ended an extraordinarily productive life in science for which he received widespread recognition, including Fellowship of The Royal Society (1966), Knighthood (1971), Companionship of Honour (1996), and many honorary degrees and prizes. He is unique, however, in having seen both universal acceptance of his work demonstrating smoking as the main cause of the most common fatal cancer in the world and the relative success of strategies to reduce the prevalence of the habit. In 1950, 80% of the men in Britain smoked but this has now declined to less than 30%.

Richard Doll qualified in medicine at St Thomas’ Hospital in 1937, but his epidemiological career began after service in the Second World War when he worked with Francis Avery Jones at the Central Middlesex Hospital on occupational factors in the aetiology of peptic ulceration. The completeness of Doll's tracing of previously surveyed men so impressed Tony Bradford Hill that he offered him a post in the MRC Statistical Research Unit to investigate the causes of lung cancer. For the representations of Percy Stocks (Chief Medical Officer to the Registrar General) and Sir Ernest Kennaway had prevailed against the then commonly held view that the marked rise in lung cancer deaths in Britain since 1900 was due only to improved diagnosis. In that now famous case–control study of Doll and Hill, the rarity of nonsmokers and the relative excess of heavy smokers among the cases compared to the controls convinced them that cigarette smoking was ‘a cause, and an important cause’ of lung cancer. A detail that he liked to recall was that the smoking habits of cases later found not to have lung cancer were similar to those of controls, another indication that the smoking differences could not be attributed simply to recall bias. A deceptively simple, but seldom appreciated, feature of the study was its definition of never-smokers as those who had never smoked on average as much as one cigarette daily for as long as a year, which was found to be unambiguous and reproducible. These were important qualities since such individuals formed the reference group, and the same definition was used in the cohort study of 40 000 British doctors that followed. This later study was unique in its regular updating of the smoking habits of participants and has continued to the present. It revealed the wide range of smoking-related diseases and, indeed, the recent 50-year follow-up found that one half of persistent smokers die as a result of the habit. The strong dose–response relationship between lung cancer and cigarette smoking, the high standard of the design and conduct of this study, and the balanced assessment of its findings in the various papers played a major part in convincing the scientific community and public health bodies around the world of a causal relationship, and in turn helped to change smoking habits. An incidental outcome of this work and of the early opposition it encountered was the development of Bradford Hill's widely used criteria for causality.

Richard Doll was one of the first epidemiologists to investigate the effects of irradiation and his follow-up with Michael Court Brown in 1957 of ankylosing spondylitis patients treated with radiation brought the first independent confirmation, after the report on atomic bomb survivors, that radiation could cause leukaemia. He described this as his favourite study; and it has continued to the present, a major source of data on the dose–response relation of radiation and cancer. Again with Court Brown, he studied British radiologists because of their repeated exposure to low doses and in 1958 reported an increased mortality from cancer among those with many years of practice early in the 20th century when high cumulative doses would presumably have resulted from the relative lack of protective procedures. More recently, his studies with Sarah Darby in relation to the fallout from atmospheric nuclear weapon testing in the 1950s and 1960s suggested that the risk of radiation-induced childhood leukaemia had not been seriously underestimated. They also studied lung cancer in relation to residential radon levels in south-west England, their results contributing to the significant positive effects found in the later meta-analysis. Non-ionising radiation was yet another of his interests and for many years (until 2002) he was chairman of the British expert group on the subject.

With Peter Armitage, he was the first to propose the multistage nature of carcinogenesis and was also responsible for classic studies of nickel refining and of asbestos. Indeed, in 1955 he was the first to show a significant excess of lung cancer among asbestos workers; identifying what is now recognised as the world's most important industrial carcinogen. He collaborated with Martin Vessey and later Valerie Beral in work on the side effects of oral contraceptives and recently studied the protective effects of alcohol on coronary heart disease.

Doll's scientific contributions were not confined to his epidemiological work. During his clinical attachment (until 1969) at the Central Middlesex Hospital, he conducted a series of therapeutic trials on peptic ulcer, including the first demonstration of the efficacy of both liquorice extract and carbenoxelone. In the 1960s he supported tropical epidemiology by seconding members of his staff to work on Burkitt's lymphoma in Africa. He had a wide influence on the progress of the science and was extensively consulted, for example, in the setting up of the International Agency for Research on Cancer (IARC). He was an early promoter of clinical trials and of cancer registries, being a long-time chairman of the MRC Leukaemia Trials Committee and an author of the first compendia of world-wide cancer incidence data in 1966 and 1970. More recently he participated actively in the important meta-analyses of breast cancer risk factors organised by Valerie Beral, and gave his support to my own work on rural population mixing as a cause of excesses of childhood leukaemia.

My own association with him dates from 1969 when he moved from the MRC Statistical Research Unit, of which he had been Director since 1961, to become Regius Professor of Medicine at Oxford. The appointment of an epidemiologist to this post was so unusual that it brought a spotlight to bear both on his department and on the subject more generally. With Malcolm Pike as his dynamic First Assistant, the department in Oxford was a hive of activity – with a constant stream of visitors from all over the world. They were heady days. If our leader had found the cause of the most common cancer, might not any of us be lucky with a less common cancer? Bruce Armstrong and Doll's well known paper in 1975 seemed to point to diet as holding keys, though sadly those hopes have largely not been fulfilled.

Richard Doll brought to the subject of epidemiology an extraordinary capacity for concentration and for work, which age (and long-haul flights) did not interrupt. Richard Peto, his closest colleague, enabled him to continue working almost every day till the end of his life; even in hospital during his final illness he did some work. Well into his 80s, working into the early hours of the morning was usual. Busy with many matters, he could still give some new question his undivided attention. But other qualities were also striking. Although recognised as a good statistician himself, he placed great importance on working with the best available in the field. He examined data with extraordinary detachment, even in an atmosphere tense with preformed opinion or other excitement; this ability also made him very effective in his frequent chairing of data monitoring committees of clinical trials. The more closely people worked with him, the more respect they had for him.

He was a master at quickly and succinctly summarising large amounts of data and was an excellent chairman, unvaryingly polite and business-like. When occasion demanded, however, he could be formidable, particularly when faced with persistent woolly thinking. Whenever possible, he was well prepared and used words carefully, saying just what he meant. His memory was exceptional, even his short-term memory in his 90s. For all his own high abilities, he was patient with colleagues. If he was expecting results, he would not mention the subject; but instead at some appropriate time, a note would be sent suggesting a meeting to talk about progress. In later years, conversation would usually turn to epidemiology where his experience was unrivalled and included personal memories of such figures as Greenwood, Stocks, Kennaway and Dorn.

While he avoided public campaigns over what actions to take about particular results, he believed strongly in communicating scientific findings to the public, and in combating misconceptions. When speaking to journalists, he did so clearly and without hesitating, often providing them with pithy quotes. He was excellent at pitching his words appropriately for his listeners and was notably effective in the courtroom.

Among his achievements at Oxford University were expanding the Medical School, a series of important professorial appointments and establishing Green College. His interests were certainly not confined to epidemiology, and included literature and theatre. On the other hand, I suspect that many subjects left him cold, though he could disguise this. He would put himself out to help both individuals and causes where he saw an injustice, but otherwise showed little interest in other people's personal lives. However, he was shrewd and observant, noticing, for example, if someone he did not know was concerned to ‘get it right’. For some people, his careful use of words and his attention to what was said to him made them self-conscious and inhibited relaxed conversation. For many, however, the correspondence between his words (spoken and written) and the essence of some issue was regularly arresting – and, I believe, contributed to the respect he commanded generally. His restraint in ‘keeping his own counsel’ could be remarkable – with the consequence that his poor opinions of certain people were seldom available for inclusion in gossip.

He had real concern for the welfare of epidemiology. For example, he was always prepared to review manuscripts for this journal, doing so succinctly and promptly! Indeed, his long association with the journal has never been equalled: his first paper in its pages appeared in 1951 (on lung cancer in relation to tobacco) and his last two in 2005 (on smoking-related cancers, and recent trends in UK cancer mortality). However, this same concern made him depressed at the recent governmental regulations that will prevent or hinder research valuable to the public health.

Apart from his contributions to science and health and his lasting effects upon many people, Richard Doll is commemorated by the existence in Oxford of Green College, and of the two flourishing units that he helped to found and in which he worked for so long: the Cancer Research UK Epidemiology Unit (under Valerie Beral) and the Clinical Trial Service Unit (under Sir Richard Peto and Rory Collins). Both are now housed in the new spacious Richard Doll Building.

**SELECTED BIBLIOGRAPHY**

The following references selected from Richard Doll's 522 publications include those referred to in the article above.

**Doctors smoking study**

Doll R, Hill AB (1954) The mortality of doctors in relation to their smoking habits. *Br Med J* **1:** 1451

Doll R, Hill AB (1956) Lung cancer and other causes of death in relation to smoking. *Br Med J* **2:** 1071

Doll R, Hill AB (1964) Mortality in relation to smoking: ten years' observations of British doctors. *Br Med J* **1:** 1399–1410, and 1460–1467

Pike MC, Doll R (1965) Age at onset of lung cancer: significance in relation to effect of smoking. *Lancet* **1:** 665–668

Doll R, Hill AB (1966) Mortality of British doctors in relation to smoking: observations on coronary thrombosis. In *Study of Cancer and Other Chronic Diseases* National Cancer Institute Monograph No. 19. Bethesda, MD

Doll R, Pike MC (1972) Trends in mortality among British doctors in relation to their smoking habits. *J Roy Coll Physicians London* **6:** 216–222

Doll R, Peto R (1976) Mortality in relation to smoking: 20 years’ observations on male British doctors. *Br Med J* **2:** 1525–1536

Doll R, Peto R (1977) Mortality among doctors in different occupations. *Br Med J* **1:** 1433–1436

Doll R, Peto R (1978) Cigarette smoking and bronchial carcinoma: dose and time relationships among regular smokers and lifelong non-smokers. *J Epidemiol Commun Health* **32:** 303–313

Doll R, Peto R, Wheatley K, Gray R, Sutherland I (1994) Mortality in relation to smoking: 40 years’ observations on male British doctors. *Br Med J* **309:** 901–911

Doll R, Peto R, Hall E, Wheatley K, Gray R (1994) Mortality in relation to consumption of alcohol: 13 years’ observations on male British doctors. *Br Med J* **309:** 911–918

Doll R (1998) Uncovering the effects of smoking: historical perspective. *Stat Methods Med Res* **7:** 87–117

Doll R, Peto R, Boreham J, Sutherland I (2000) Smoking and dementia in male British doctors: prospective study. *Br Med J* **320:** 1097–1102

Peto R, Darby S, Deo H, Silcocks P, Whitley E, Doll R (2000) Smoking, smoking cessation and lung cancer in the UK since 1950. *Br Med J* **321:** 323–332

Doll R, Peto R, Boreham J, Sutherland I (2004) Mortality in relation to smoking: 50 years’ observations on male British doctors. *Br Med J* **328:** 1519–1528

Doll R, Peto R, Boreham J, Sutherland I (2005) Mortality in relation to alcohol consumption: a prospective study among British doctors. *Int J Epidemiol* **34:** 199–204

Doll R, Peto R, Boreham J, Sutherland I (2005) Mortality from cancer in relation to smoking: 50 years observations on British doctors. *Br J Cancer* **92:** 426–429

**Other smoking**

Doll R, Hill AB (1950) Smoking and carcinoma of the lung. *Br Med J* **2:** 739

Daff ME, Doll R, Kennaway EL (1951) Cancer of the lung in relation to tobacco. *Br J Cancer* **5:** 1–20

Doll R, Hill AB (1952) A study of aetiology of carcinoma of the lung. *Br Med J* **2:** 1271

Doll R, Hill AB, Kreyberg L (1957) The significance of cell type in relation to the aetiology of lung cancer. *Br J Cancer* **11:** 43–48

Doll R, Hill AB, Gray PG, Parr EA (1959) Lung cancer mortality and the length of cigarette ends: an international comparison. *Br Med J* **1:** 322–325

Peto R, Lopez AD, Boreham J, Thun M, Heath Jr C, Doll R (1996) Mortality from smoking worldwide. *Br Med Bull* **52**(1): 12–21

**Radiation**

***Ankylosing spondylitis study***

Court Brown WM, Doll R (1957) *Leukaemia and aplastic anaemia in patients irradiated for ankylosing spondylitis*. Med Res Council Spec Rep Ser No. 295. London: HMSO

Weiss HA, Darby SC, Doll R (1994) Cancer mortality following X-ray treatment for ankylosing spondylitis. *Int J Cancer* **59:** 327–338

Weiss HA, Darby SC, Fearn T, Doll R (1995) Leukaemia mortality following X-ray treatment for ankylosing spondylitis. *Radiat Res* **142:** 1–11

***British Radiologists Study***

Court Brown WM, Doll R (1958) Expectation of life and mortality from cancer among British radiologists. *Br Med J* **2:** 181–187

Berrington A, Darby SC, Weiss HA, Doll R (2001) 100 years of observation on British radiologists: mortality from cancer and other causes 1897–1997. *Br J Radiol* **74:** 507–519

***Other***

Forman D, Cook-Mozaffari P, Darby S, Davey G, Stratton I, Doll R, Pike M (1987) Cancer near nuclear installations. *Nature* **329:** 499–505

Darby S, Olsen JH, Doll R, Thakrar B, Brown P de N, Storm HH, Barlow L, Langmark F, Teppo L, Tulinius H (1992) Trends in childhood leukaemia in the Nordic countries in relation to fallout from atmospheric nuclear weapons testing. *Br Med J* **304:** 1005–1009

Doll R, Evans HJ, Darby SC (1994) Paternal exposure not to blame. *Nature* **367:** 678–680

Doll R (1995) Hazards of ionizing radiation: 100 years of observations on man. *Br J Cancer* **72:** 1339–1349

Doll R, Wakeford R (1997) Risk of childhood cancer from fetal irradiation. *Br J Radiol* **70:** 130–139

Darby S, Hill D, Doll R (2005) Radon in homes and risk of lung cancer: collaborative analysis of individual data from 13 European case–control studies. *Br Med J* **330:** 223–227

**Occupational**

Doll R (1955) Mortality from lung cancer in asbestos workers. *Br J Ind Med* **12:** 81–86

Doll R (1958) Cancer of the lung and nose in nickel workers. *Br J Ind Med* **15:** 217–223

Doll R, Fish REW, Gammon EJ, Gunn W, Hughes GO, Tyrer FH, Wilson W (1965) Mortality of gasworkers with special reference to cancers of the lung and bladder, chronic bronchitis, and pneumoconiosis. *Br J Ind Med* **22:** 1–12

Doll R (1984) In: Sunderman FW (Ed) *Nickel Exposure: A Human Health Hazard* IARC Scientific Publication No. 53, pp 3–21

Doll R, Peto J (1985) Asbestos: effects on health of exposure to asbestos. A report to the Health and Safety Commission, London: HMSO

**Diet, immune, HIV, childhood leukaemia etc.**

Armstrong B, Doll R (1975) Environmental factors and cancer incidence and mortality in different countries, with special reference to dietary practices. *Int J Cancer* **15:** 617–631

Kinlen LJ, Sheil AGR, Peto J, Doll R (1979) Collaborative United Kingdom–Australasian study of cancer in patients treated with immunosuppressive drugs. *Br Med J* **2:** 1461–1466

Franceschi S, Doll R, Gallwey J, LA Vecchia C, Peto R, Spriggs AI (1983) Genital warts and cervical neoplasia: an epidemiological study. *Br J Cancer* **48:** 621–628

Doll R (1987) Major epidemics of the 20th century: from coronary thrombosis to AIDS. *J Roy Stat Soc, Ser A* **150:** 373–395

Darby SC, Rizza CR, Doll R, Spooner RJD, Stratton IM, Thakrar B (1989) Incidence of AIDS and excess of mortality associated with HIV in haemophiliacs in the United Kingdom: report on behalf of the directors of haemophilia centres in the United Kingdom. *Br Med J* **298:** 1064–1068

Doll R (1999) The Seascale cluster: a probable explanation. *Br J Cancer* **81:** 3–6

Kinlen L, Doll R (2004) Population mixing and childhood leukaemia: Fallon and other US clusters. *Editorial. Br J Cancer* **91:** 1–3

**Peptic ulcer**

Doll R, Jones FA, Buckatzsch MM (1951) Occupational factors in the aetiology of gastric and duodenal ulcers. Med Res Council Spec Rep Ser No. 276. London: HMSO

Doll R, Hill ID, Hutton C, Underwood DJ (1962) Clinical trial of a triterpenoid liquorice compound in gastric and duodenal ulcer. *Lancet* **2:** 793–796

Doll R, Hill ID, Hutton CF (1965) Treatment of gastric ulcer with carbenoxolone sodium and oestrogens. *Gut* **6:** 19–24

**Hormonal**

Vessey MP, Doll R (1968) Investigation of relation between use of oral contraceptives and thromboembolic disease. *Br Med J* **2:** 199–205

Vessey MP, Doll R (1976) Is ‘the pill’ safe enough to continue using? *Proc Roy Soc London B* **195:** 69–80

Vessey MP, Doll R, Jones K, McPcherson K, Yeates D (1979) An epidemiological study of oral contraceptives and breast cancer. *Br Med J* **1:** 1757–1760

Collaborative Group on Hormonal Factors in Breast Cancer – writing committee: Beral V, Bull D, Doll R, Peto R, Reeves G. Papers on hormonal contraceptives (*Lancet* 1996; **347:** 1713–1727); Hormone replacement therapy (*Lancet* 1997; **350:** 1047–1059); Alcohol. (*Br J Cancer* 2002; **87:** 1234–1245); Abortion (*Lancet* 2004; **363:** 1007–1016); Breast Feeding (*Lancet* 2002; **360:** 187–195); Hormonal factors (*Lancet* 2001; **358:** 1389–1399)

**General and other**

Armitage P, Doll R (1954) The age distribution of cancer and a multi-stage theory of carcinogenesis. *Br J Cancer* **8:** 1–12

Doll R, Payne P, Waterhouse J (eds)(1966) *Cancer Incidence in Five Continents*. Berlin: UICC, Springer-Verlag

Doll R, Cook P (1967) Summarizing indices for comparison of cancer incidence data. *Int J Cancer* **2:** 269–279

Doll R (1967) *Prevention of Cancer: Pointers from Epidemiology*. London: Nuffield Provincial Hospitals Trust

Cook P, Doll R, Fellingham SA (1969) A mathematical model for the age distribution of cancer in man. *Int J Cancer* **4:** 93–112

Doll R (1977) Strategy for detection of cancer hazards to man. *Nature* **265:** 589–596

Doll R (1978) An epidemiological perspective of the biology of cancer. *Cancer Res* **38:** 3573–3583

Doll R, Peto R (1981) The causes of cancer. Quantitative estimates of avoidable risks of cancer in the United States today. *J-Natl Cancer Inst* **66:** 1195–1308 (published by Oxford University Press)

Collins R, Doll R, Peto R (1992) Ethics of clinical trials. In *New Treatments for Cancer: Practical, Ethical, and Legal Problems* CJ Williams (Ed) New York: John Wiley & Sons

Doll R, Peto R (2001) Rights involve responsibilities for patients (letter). *Br Med J* **322:** 730

Doll R (2001) Research will be impeded. *Br Med J* **323:** 1421–1422

Doll R (2002) Proof of causality: deduction from epidemiological observation. *Persp Biol Med* **46:** 499–515

Doll R, Boreham J (2005) Recent trends in cancer mortality in the UK. *Br J Cancer* **92:** 1329–1335